# Male group size, female distribution and changes in sexual segregation by Roosevelt elk

**DOI:** 10.1371/journal.pone.0187829

**Published:** 2017-11-09

**Authors:** Leah M. Peterson, Floyd W. Weckerly

**Affiliations:** Department of Biology, Texas State University, San Marcos, TX, United States of America; University of Missouri Columbia, UNITED STATES

## Abstract

Sexual segregation, or the differential use of space by males and females, is hypothesized to be a function of body size dimorphism. Sexual segregation can also manifest at small (social segregation) and large (habitat segregation) spatial scales for a variety of reasons. Furthermore, the connection between small- and large-scale sexual segregation has rarely been addressed. We studied a population of Roosevelt elk (*Cervus elaphus roosevelti*) across 21 years in north coastal California, USA, to assess small- and large-scale sexual segregation in winter. We hypothesized that male group size would associate with small-scale segregation and that a change in female distribution would associate with large-scale segregation. Variation in forage biomass might also be coupled to small and large-scale sexual segregation. Our findings were consistent with male group size associating with small-scale segregation and a change in female distribution associating with large-scale segregation. Females appeared to avoid large groups comprised of socially dominant males. Males appeared to occupy a habitat vacated by females because of a wider forage niche, greater tolerance to lethal risks, and, perhaps, to reduce encounters with other elk. Sexual segregation at both spatial scales was a poor predictor of forage biomass. Size dimorphism was coupled to change in sexual segregation at small and large spatial scales. Small scale segregation can seemingly manifest when all forage habitat is occupied by females and large scale segregation might happen when some forage habitat is not occupied by females.

## Introduction

Sexual segregation, or the differential use of space by males and females, is ubiquitous among size-dimorphic vertebrates [1]. The drivers of this behavior are of interest because spatial partitioning affects individual response to environmental change, demography and population dynamics [2–4]. The degree of sexual segregation in mammals often varies seasonally, reflecting a lower degree of segregation during the mating season and a higher degree of segregation during parturition and lactation [5, 6]. These seasonal patterns are often attributed to the intersexual difference in requirements for achieving reproductive success [7]. The persistence of sexual segregation beyond parturition and mating seasons infers that what occurs outside of the times of reproductive events must also be influential [8, 9].

Size dimorphism has evolved from sexual selection in polygynous ungulate species [10, 11]. Often, adult males of these species are larger in body size than female conspecifics, because large males usually have a competitive advantage over smaller males for breeding opportunities [12]. In contrast, females grow to a sufficient body size to reproduce and to increase the probability of survival through seasons of food limitations, and thereafter, reproductive success depends on maternal nutrition and body condition [13, 14]. The different body sizes probably led to intersexual differences in digestive capability and forage acquisition [15, 16]. Because body sizes and the means to achieve reproductive success differ between males and females, partitioning of resources and sexual segregation might manifest for a variety of reasons and at multiple spatial scales [10, 17].

Perhaps in accordance with larger body sizes, adult males are socially dominant to females in gregarious ungulate species. These individuals display more intense aggression, engaging in and winning the majority of aggressive bouts with females and younger males [18–21], therefore, sexual segregation may be influenced by adult male ungulates as a means of females avoiding aggression. Ungulate populations can exhibit mixed-sex aggregations or single-sex groups [22–24]. Females more often exist in socially bonded groups [25], while males display a lower degree of social bonding and are solitary or occur in small-sized groups comprised of other adult males [26]. While aggressive bouts between females may be costly, male–male aggression has been shown to be beneficial to reproductive success of males that win aggressive interactions [12]. When adult males are in female groups, the rate of female–female aggression increases with increasing number of males, since male–male competition makes females more likely to approach unfamiliar or dominant females while avoiding males [27]. Consequently, a group of females is more likely to walk away if a large group of males approaches than if the approaching group of males is small in size [28]. According to this female avoidance hypothesis, a prediction is that male group size will be positively associated with sexual segregation at the group level. Segregation at the group level often occurs within habitat or at a small spatial scale, so-called social segregation.

At the larger spatial scale of habitat segregation, the different foraging niches and life histories of females and males might also be associated with sexual segregation. Males have a wider forage niche than females which results in consumption of a greater variety of forages and use of habitats [17, 29]. Associated with the wider forage niche of males is that males tend to show less site tenacity than females [30]. Furthermore, females, burdened with parental care, tend to select habitat that possesses fewer lethal risks [31, 32]. Males, on the other hand, with a large body size, greater forage demands, and no burdens from parental care might use forage habitat with greater lethal risks [33]. An outcome of these behaviors can be described as the vacant forage habitat, such that if females vacate forage habitat, then males should use that habitat. The shift in use of habitat should result in a change in habitat segregation.

Of course, a change in sexual segregation might simply be associated with a change in forage abundance. All else being equal, larger males require a greater forage intake than the smaller females. Thus, the prediction from the forage abundance hypothesis is that males should forage in habitat with greater amounts of forage biomass.

The aim for this study was to investigate what was coupled to changes in sexual segregation across 21 years in a population of Roosevelt elk (*Cervus elaphus roosevelti*) in north, coastal California, USA. In particular, we examined the predictions of each of three hypotheses. The first—did social segregation positively associate with male-only group sizes? Secondly—did habitat segregation change when a forage habitat was vacated by females, and third—was a change in habitat segregation associated with changes in forage abundance? The findings of this study should have implications for understanding the connections between social and habitat segregation outside of seasons of mating and parturition.

## Methods

### Ethics statement

Prior to the initiation of the study, it was realized that no animals would be handled or approached to interfer with and disrupt animal activity. Nonetheless, all animal research was reviewed and approved by the Texas State University Institutional Animal Care and Use Committee (IACUC), permit numbers 04-046876343F, 07-1106-07, 1035_1112_31, 1019_1031_23, 20168174611.

### Study area

**Fig 1:**
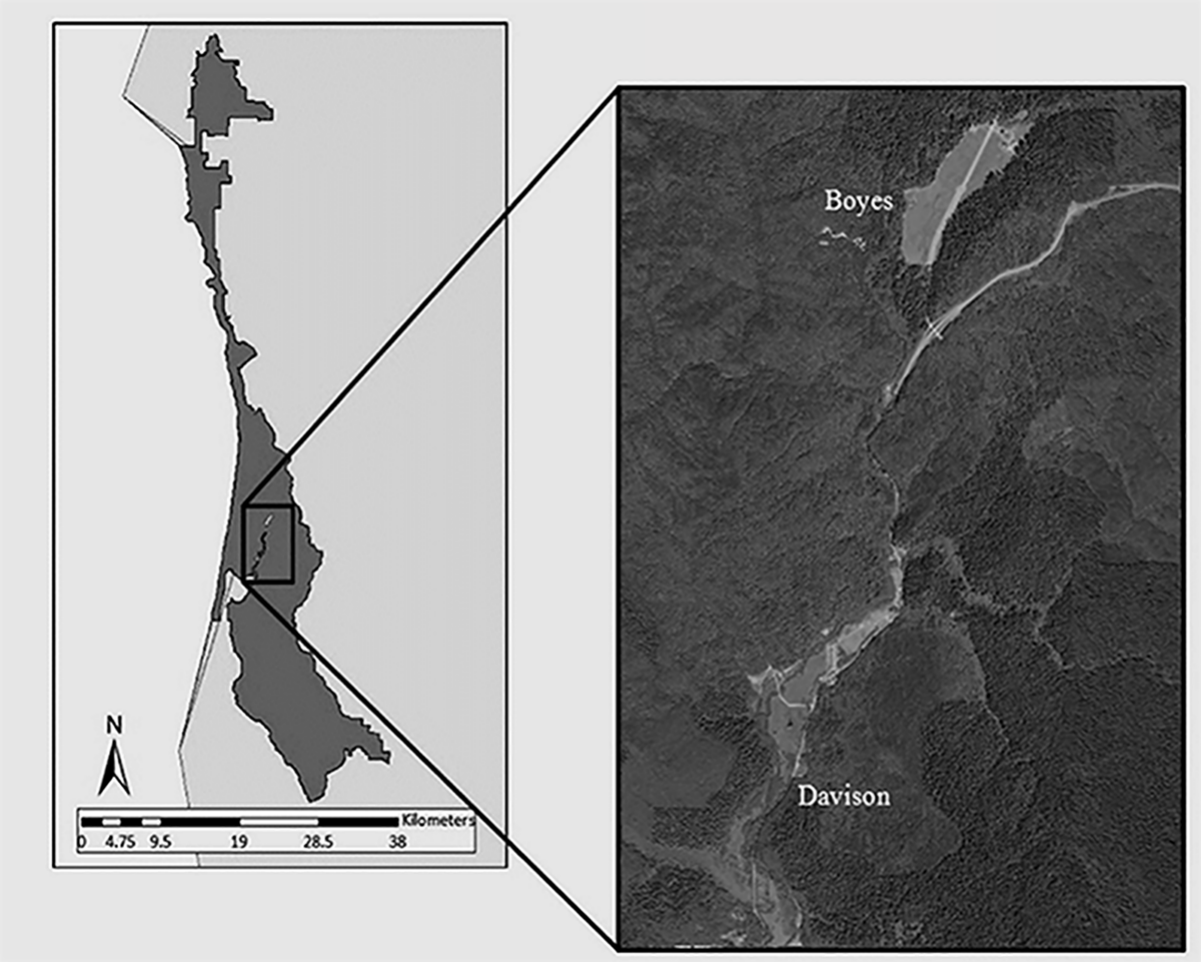
Aerial photograph of the two meadow complexes in the Prairie Creek Drainage in Redwood National and State Parks. Visible to the south and east of Boyes meadow is U.S. Hwy 101.

This study was conducted in the Prairie Creek Drainage (41°20’N, 12400B00302’W) of the Redwood National and State Parks, Humboldt County, California, USA (**Fig 1**). This region of California exhibits a maritime climate with cool summers and rainy winters. The average annual rainfall was approximately 1,650 mm with daytime temperatures in the low teens (Celsius) in winter and high teens in summer [34]. The Prairie Creek Drainage consisted of two distinct meadow complexes, Boyes and Davison, that were roughly 3.0 km apart. The Boyes meadow complex was comprised of the large Boyes meadow and meadows along U.S. Highway 101 to the south and east of Boyes meadow, totaling 70 ha of meadows. Vegetation in the meadows were forbs, as well as perennial and annual grass species such as California oatgrass (*Danthonia californica*), redtop (*Agrostis gigantea*), and softchess (*Bromis hordeaceus*). Boyes meadow also had Bracken fern (*Pteridium aquilinum*) and California blackberry (*Vitus ursinus*). At least 66 percent of Boyes meadow was burned in September or October 1996, 2000, 2005, 2006, 2008, 2013 and 2016. The Davison meadows totaled 51 ha and were covered by perennial and annual grasses many of which were also found in the Boyes meadow [27]. Reed canary grass (*Phalaris arundinacea*) became prevalent in the Davison meadows shortly after 2000. Some forbs (*Ranunculus*, *Trifolium*) were also present in Davison meadows. Unlike Boyes meadow, Davison meadows have not been burned [35]. Both meadow complexes were surrounded by old-growth and second-growth coastal redwood (*Sequoia sempervirens*)-conifer forests. Common conifer species included Douglas fir (*Pseudotsuga menziesii*), Sitka spruce (*Picea sitchensis*), and western hemlock (*Tsuga heterophylla*). The elk residing in this drainage were non-migratory and not legally hunted. Natural predators included coyote (*Canis latrans*), bobcat (*Lynx rufus*), mountain lions (*Puma concolor*), and black bear (*Ursus americanus*).

### Data collection

Previous observations of the elk in Prairie Creek Drainage indicate that males and females are mostly aggregated during the rut from late August to October and they are mostly segregated during parturition in May and June [9, 36]. Furthermore, predation may also influence sexual segregation, either during spring and summer when newborn calves are vulnerable or during fall when exhausted, post-rut males are vulnerable [37, 38]. To reduce the influence that reproductive or predation pressures might have on sexual segregation, data were collected each January from 1997 to 2017. Population surveys were conducted each year by driving on a predetermined route through the study area [39]. Ten surveys (beginning at daybreak and lasting 1.75 hours) were conducted each year with the exception of 1998 and 1999, when only 5 surveys were completed. Since the elk were habituated to human presence, observations were always collected within 200 meters of the elk with either the natural eye or binoculars. During observations, groups were typified into two categories: adult male-only groups or female groups, which were comprised of adult females, sub-adult (1.5-year-old) males and juveniles (<1.5-year-old). Sub-adult females were included with adult females due to the ostensible lack of indistinguishable morphologies. Also, one or a few (<5) adult males could be associated with females. The latter group type was characterized as a “female” group, rather than “mixed-sex” because female elk exhibit high degrees of social bonding and group stability [25], yet adult males often do not coexist with female groups for long periods of time [8, 27, 38, 40]. Observed elk were recorded as marked or unmarked, with marked individuals identified by ear-tags or morphological anomalies [39]. The majority of adult males were marked and at least one elk from every observed female herd was marked. Thus, the Boyes and Davison herds were distinguishable from one another. Total population abundance (adult males: adult female, sub-adults and juveniles) and adult male abundance were estimated in one of two ways across years. Abundances were estimated using Bowden’s mark-resight estimator when there were unmarked males detected during surveys [41]. Compared to males, females had high (>80%) sighting probabilities across the 5 or 10 surveys [3]. Most of the sighting heterogeneity came from males and not females. When all males detected across all surveys were marked, we used the highest count in that year as the estimate of abundance.

Forage biomass was estimated in January, from 2005 to 2017, in quarter-meter plots placed along transects dispersed throughout seven sectors (sub-meadows) in the Davison meadow complex and four sectors in the Boyes meadow complex [3]. Sectors were delineated to insure that all parts of meadow complexes were measured. Transects were randomly placed in sectors. There were 550 plots measured in 2005, and 570 measured in the remaining years. Vegetation height was measured to the nearest centimeter at eight equidistant locations within the plot. The cover of palatable and green grasses, forbs, and shrubs were estimated using Daubenmire coverage classes: 0–5%, 6–25%, 26–50%, 51–75%, 76–95%, and 96–100% coverage [42]. From 2005 to 2007, clippings down to ground level of palatable grasses, forbs and shrubs were sorted and dried at 60°C for 48 hrs [3]. Multiple regressions with predictors of plant heights averaged across the eight equidistant measurements and coverage classes predicted dried biomass of grasses (*r*^2^ = 0.84, *F*_7, 122_ = 97.1, *P* < 0.001) as well as forbs and shrubs (*r*^2^ = 0.33, *F*_2, 93_ = 24.9, *P* < 0.001). These regressions were then used to estimate forage biomass in each of the 550 or 570 plots.

Forage biomass was summarized in two ways, per hectare of meadow in each complex, and on a per capita basis. We estimated per hectare forage biomass because it probably indexed bite size and forage intake [3]. Most forage biomass in the Prairie Creek Drainage ranged from 100 to 500 ha in meadows in January. Across the spectrum of forage biomasses (100–7000 kg^.^ ha^-1^) that elk might experience across seasons, forage in January is on the scarce end. When forage biomass is limited, bite size and forage intake display a positive relationship with biomass [43]. Per capita forage biomass was estimated to assess grazing pressure [3, 44]. This metric was calculated by summing forage biomass across sectors and dividing by elk abundance in each meadow complex (kg^.^ elk^-1^).

### Analyses

The sexual segregation and aggregation statistic (SSAS) was calculated to estimate sexual segregation at the level of the group (small-scale) and meadow complex (large-scale) using the chi-square statistic. A group was defined as solitary elk or aggregations of elk displaying coordinated activity within 50 m of one another [25]. The SSAS was calculated using the formula developed by [45]:

$$SSAS=1-\frac{N}{XY}\sum_{i=1}^{k}\frac{X_{i}Y_{i}}{N_{i}}$$

where *N* was the sum of total encountered males (*X*) and total encountered females (*Y*). For estimating segregation at the level of the group, the data were summed from *k* groups with *X*_*i*_ males and *Y*_*i*_ females in the *i*th group with a group size of *N*_*i*_. To estimate segregation by meadow complex, the data was organized from *k* observations of female and male elk in the two meadow complexes across the 5 or 10 surveys. If males and females were randomly associated, then the estimated SSAS from the observed data fell within the 2.5^th^ and 97.5^th^ percentiles of 10,000 randomized SSAS values (95% confidence band). The randomization or permutation procedure to test for segregation is recommended because the estimated SSAS is influenced by the distribution of group sizes and population sex ratio, both of which often vary across years [45]. Estimates of SSAS can range between 0 and 1, inclusively. The population would be regarded as sexually segregated or aggregated if the SSAS was greater than or less than the 95% confidence band, respectively. Biologically, the SSAS can be used to determine whether the sex ratio at the level of the group or meadow complex deviates from the population sex ratio [45]. Both SSASs were estimated for each year of the study to determine which years the population of elk was sexually segregated, aggregated, or randomly associated across groups, as well as between meadow complexes.

Typical group size (TGS) was estimated to reflect a male-only group size for each year because it was explicitly formulated as an animal-centered measure of gregariousness, as opposed to a generic mean group size which can be inordinately influenced by solitary animals [46]. Male TGS was estimated by summing the square of observed sizes of male-only groups divided by the sum of observed sizes of male-only groups. This metric included solitary males [47].

To assess the influence of TGS on segregation at the group level we estimated correlations between TGS and SSAS interpretations at the group level (group-SSAS), as well as between population abundance and group-SSAS interpretations. The group-SSAS were interpreted as either random association or sexual segregation (coded 0 or 1, see Results). We also estimated partial correlation coefficients between group-SSAS interpretations and TGS, controlling for population abundance; and between group-SSAS and population abundance, controlling for TGS [48]. Sexual segregation might be density-dependent [17, 32]. Furthermore, typical group size is related to male abundance, and male abundance can be correlated to population abundance [49]. Thus an association between TGS and group-SSAS interpretations might be spurious. Probability values of correlation coefficients were estimated from 10,000 randomizations or permutations of the data. The type I error rate can be estimated more accurately from randomized data for correlation coefficients [50].

We conducted a Fisher’s exact test to assess whether females vacating a meadow complex was associated with change in meadow complex SSAS (meadow-SSAS) interpretations [48]. Meadow-SSAS were interpreted as aggregated, random, or segregated (coded 0, 1, or 2, respectively). It was also conceivable that meadow-SSAS interpretations were correlated with TGS since small groups of elk tend to range more widely and use more forested habitat than large groups, presumably because forage demands are less [29]. We estimated the correlation between the meadow-SSAS interpretations and TGS; as well as a correlation between meadow-SSAS and TGS, controlling for females vacating the Boyes meadow complex (0 –vacant, 1 –occupied).

We fit four linear mixed models to assess the effects of time, females vacating Boyes meadow and segregation on forage biomass. The first model estimated the influence of each year and meadow complex on forage biomass (kg^.^ ha^-1^). It is likely that forage biomass differs between meadow complexes as well as across years for reasons besides elk herbivory. For this model, year was categorical. A second model estimated whether there was a linear trend across years in forage biomass for each meadow complex. Elk abundance tended to decline across the time series (see results) and there might have been a decline in forage abundance across years as well. In this model year was coded as a numeric predictor. The third model estimated whether years before and after females vacated one of the meadow complexes influenced forage biomass. The fourth model assessed whether changes in group level sexual segregation coincided with changes in forage biomass. Years when groups were sexually segregated or randomly associated, based on group-SSAS interpretations, were coded as a categorical predictor. In every model, an interaction between meadow complex and the other predictor was estimated. Linear mixed-effects models had sector as the random factor and an intercepts random effect [51]. To evaluate which model fit the data we used Bayesian Information Criterion (BIC). We chose BIC over the more popular Akaike Information Criterion (AIC) because the first model was much more complex than remaining models. The AIC tends to favor more complex models [52]. The model selection analysis was conducted on models estimated with maximum likelihood estimators but parameter estimates of the selected model were estimated with restricted maximum likelihood estimation [14]. We also estimated the marginal and conditional *R*^2^ for each model [53]. The marginal *R*^2^ estimated the variation in the response variable accounted for by the fixed effects and the conditional *R*^2^ estimated the variation in the response variable accounted for by the fixed and random effects. To assess if per capita elk forage differed between meadow complexes we estimated a generalized least squares model. The response variable was the estimated per capita forage biomass with meadow complex as the predictor and a lag 1 autocorrelation function [54].

## Results

**Fig 2:**
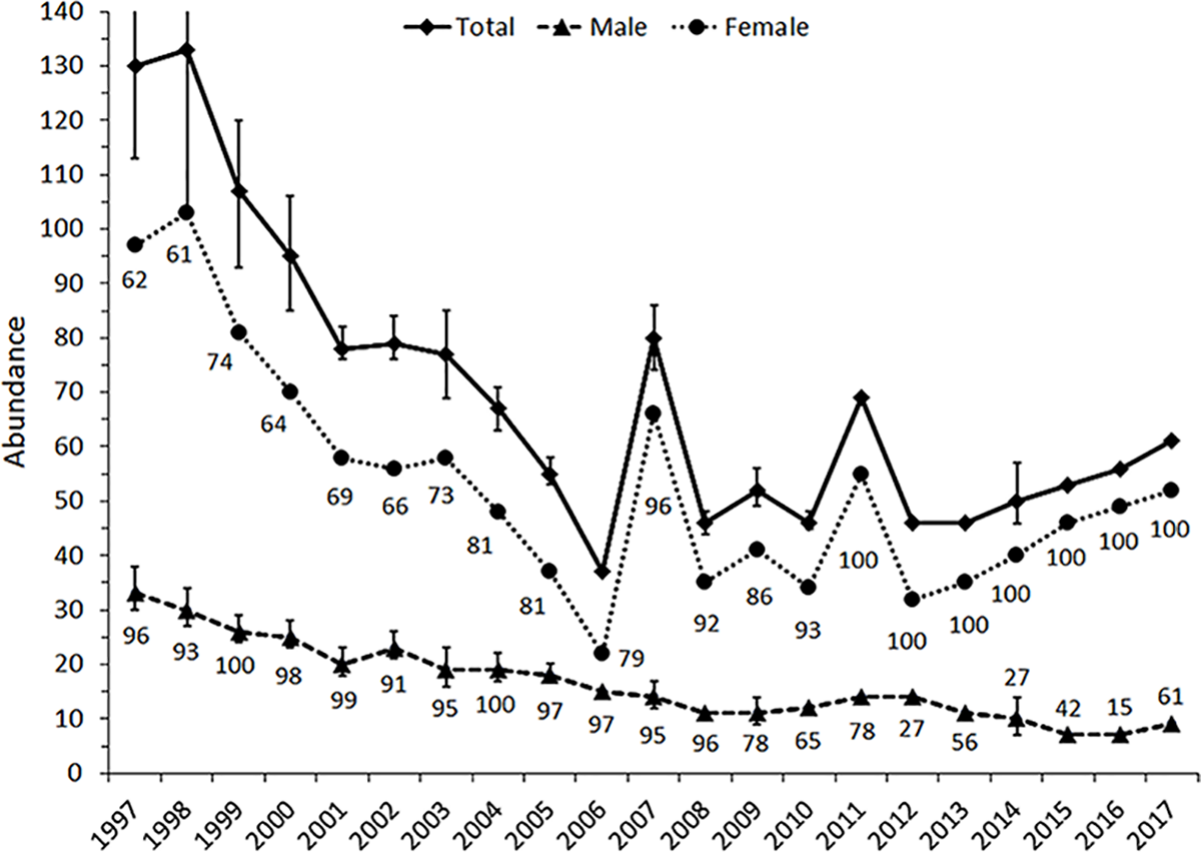
Abundances of the total population, female (included juveniles and sub-adult males), and adult male Roosevelt elk (*Cervus elaphus roosevelti*) in Prairie Creek Drainage, Redwood National and State Parks, California, USA, from 1997 to 2017. Percentages of females and males encountered in the Davison meadow complex are presented by the respective female and male lines.

The population peaked early in the time series at 130 to 133 elk (**Fig 2**). After 1998 the population declined steadily to 37. Thereafter, the population increased steadily (with two spikes) to an abundance of 61 in 2017. The abrupt increases in 2007 and 2011 were from the Levee herd inhabiting the southern part of the Davison meadow complex [3]. The Levee herd usually occupied meadows to the south of the Prairie Creek Drainage. The temporal changes in female abundance were concomitant to those of the total population, while male abundance declined across the 21 years. In 1997, the estimated male abundance was 33, and in 2015 and 2016 there were seven males. In general, the percentage of the female population encountered in Davison complex increased across the time series. There were two female herds (Boyes and Davison) sighted every year between 1997 and 2010. From 2011 to 2017, there were no females sighted in the Boyes meadow complex. Males, however, were sighted more frequently in the Davison meadow complex early in the time series. Between 1997 and 2008, 91 percent or more of males were encountered in the Davison and the remaining 9 percent or less were sighted in the Boyes meadow complex. Whereas after 2008 the percentage of males that were encountered in the Davison meadow complex dropped to 78 percent or less.

**Fig 3:**
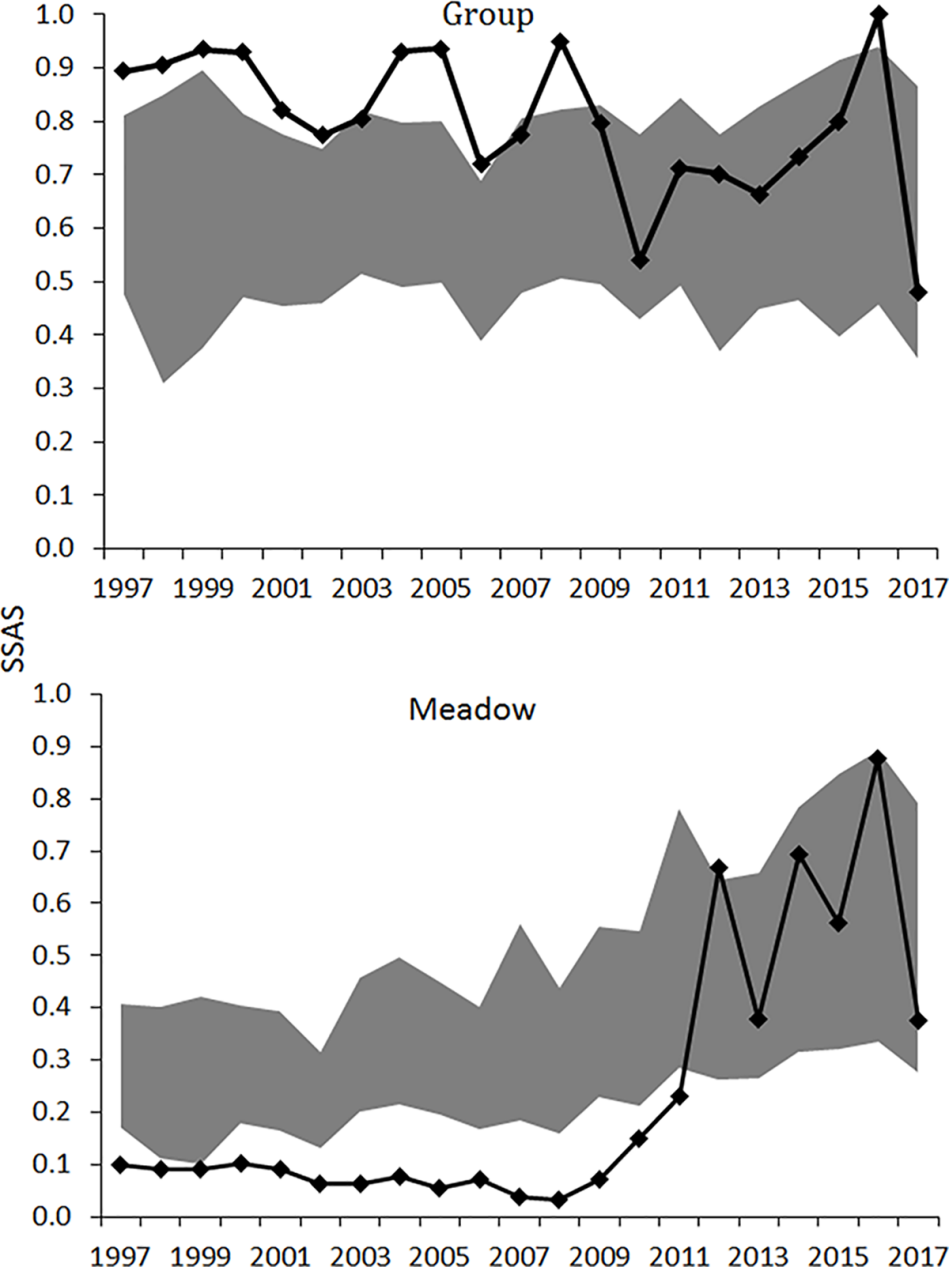
Group and meadow complex SSASs, or the sexual segregation and aggregation statistics, (solid, black line and diamonds) for Roosevelt elk (*Cervus elaphus roosevelti*) from 1997 to 2017 in Prairie Creek Drainage, Humboldt County, California, USA. The gray 95% confidence band indicates when males and females were randomly associated whereas SSAS values above the 95% confidence band indicate sexual segregation and SSAS values blow the band indicate sexual aggregation. In each year, there were 17–59 groups or solitary elk encountered.

The group-SSAS estimates indicated that elk were segregated in eight of the twelve years between 1997 and 2008 (**Fig 3**). After 2008, there was only one year when female and male elk were segregated by group. In the remaining years elk were randomly associated in groups.

The patterns differed for the meadow-SSAS interpretations (**Fig 3**). From 1997 to 2011, females and males were aggregated in the Davison meadow complex. In 2011 females and males were habitat segregated, females in Davison meadows and males in the Boyes meadow complex. Afterwards, females and males were randomly associated in the two meadow complexes.

Typical group size ranged from 2.7 to 13.2 and averaged 6.7; TGS was larger early in the time series and coincided with male abundance across the time series (*r* = 0.90, *P* < 0.001). The group-SSAS interpretation was associated with TGS (*r* = 0.70, *P* < 0.001) and marginally associated with total abundance (*r* = 0.42, *P* = 0.058). When total abundance was partially controlled, the correlation between TGS and group-SSAS was 0.65 (*P* = 0.007). Using TGS as the partial variable, there was no correlation between total abundance and group-SSAS (*r* = -0.23, *P* = 0.358). In 2012 TGS was 7.8 yet females and males were separated in different meadow complexes (**Fig 3)**. Because the opportunity for females and males to encounter each other seemed remote we recalculated the partial correlations excluding the data collected in 2012. When total abundance was the partial variable the correlation between TGS and group-SSAS was 0.76 (*P* < 0.001). When TGS was the partial variable, there was a marginal correlation between total abundance and group-SSAS (*r* = -0.45, *P* = 0.069). Typical group size was more strongly associated with group-SSAS interpretations than total abundance.

A Fisher’s exact test indicated an association between meadow-SSAS interpretations and whether the Boyes female herd was present or absent (*P* < 0.001). Before 2011, the Boyes herd was present and females and males remained aggregated in the Davison meadow complex until 2011. In 2012, females and males displayed habitat segregation, thereafter females and males were randomly associated across the two meadow complexes. Typical group size, however, was not associated with interpretations of the meadow-SSAS (*r* = -0.32, *P* = 0.152). Also, when controlling for whether the Boyes female herd was present or absent, no correlation between TGS and meadow-SSAS interpretations was evident (*r* = 0.27, *P* = 0.272). The meadow-SSAS interpretation was associated with females vacating the Boyes meadow complex.

**Fig 4:**
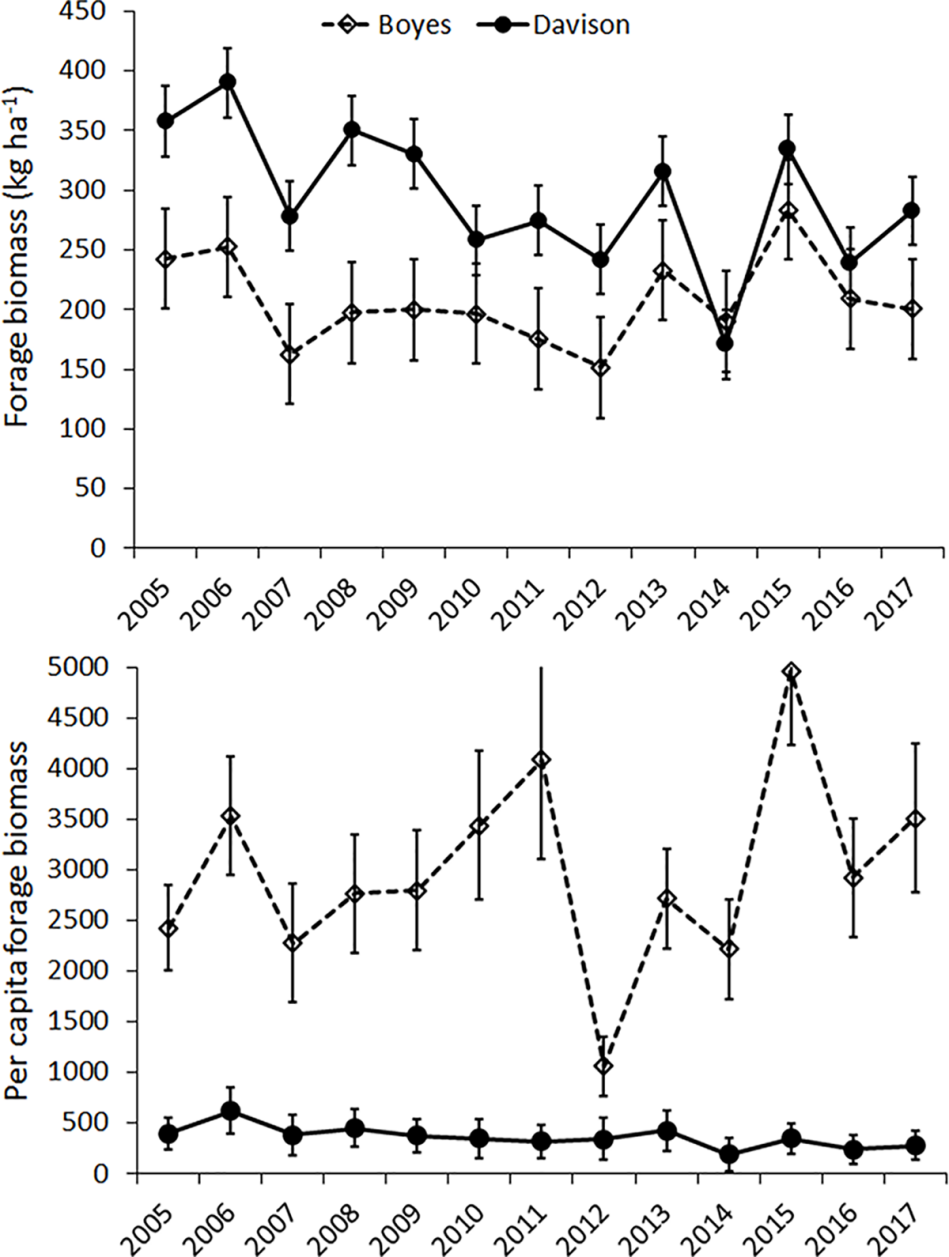
Estimated means and 1 standard error bars of per ha and per capita (total forage biomass^.^ total elk abundance^-1^) forage biomass from 2005 to 2017 in the two meadow complexes in the Prairie Creek Drainage, Humboldt County, California, USA.

The model selection analysis indicated that linear trend over the years, females vacating the Boyes meadow complex, or the group segregation patterns did not influence forage biomass as much as the model estimating per hectare forage biomass by meadow complex and year (**Table 1**). Although the fixed factors in every model did not account for much variation in forage biomass, the marginal *R*^2^ of the selected model explained 5–6 percent more variation than remaining models. For the selected model, variance across sectors (random effect) was 3.6 and the residual variance or variance within sectors was 11.4. Forage biomass per hectare was noticeably greater in the Davison meadow complex in most years between 2005 and 2017 (**Fig 4**). When forage biomass was expressed on a per capita basis, the trends in each meadow complex were strikingly different from per ha forage biomass. There appeared to be lower per capita forage, or greater elk grazing pressure, in the Davison meadow complex and greater per capita forage, or lighter elk grazing pressure, in the Boyes meadow complex (*F*_1,24_ = 91.07, *P* < 0.0001).

**Table 1:** Summary of a Bayesian Information Criterion (BIC) model selection analysis estimating forage biomass in Boyes and Davison meadow complexes from 2005 to 2017. Presented for each linear mixed effects model is the number of parameters estimated (*K*), log-likelihood (*LL*), delta (*Δ*), or difference in BIC between a model and the model with the smallest BIC, and the marginal $\left(R_{m}^{2}\right)$ and conditional $R_{c}^{2}$ values. Year was a categorical predictor and year linear estimated a linear trend across years. Years when groups were sexually segregated or comprised of randomly associated females and males was labeled group seg.

Model predictors	*K*	*LL*	*Δ*	$R_{m}^{2}$	$R_{c}^{2}$
Meadow, year, meadow X year	28	-19668.2	0.0	0.14	0.35
Meadow, year linear, meadow X year linear	6	-20006.0	479.4	0.09	0.32
Meadow, Boyes herd, meadow X Boyes herd	6	-20012.6	492.6	0.09	0.30
Meadow, group seg, meadow X group seg	6	-20018.8	504.9	0.08	0.29

## Discussion

Some studies of sexual segregation examine factors driving the seasonality of the phenomenon [32, 55]. Yet, the finding from such studies can be constrained by seasonal changes in life-history strategies. Using a 21-year-long data set, we detected changes in sexual segregation at a time of the year when complications of seasonal reproductive demands were presumably reduced. Our findings were consistent with the female avoidance hypothesis for small spatial scale—social or group level segregation—and the vacated female hypothesis for large-scale—habitat segregation. Before 2011, both meadow complexes were occupied by female herds and most males used the Davison meadow complex. Between 2005 and 2010, the Davison meadow complex also had more forage on a per hectare basis than the Boyes meadow complex. Particularly early in the time series, females and males in the Davison meadow complex were abundant and in close proximity. One potential mechanism to keep females and males segregated at a small spatial scale was females avoiding larger groups of males [27], which may explain why there was a moderate positive correlation between TGS and group-SSAS interpretations. This correlation appeared to be stronger when we excluded data collected in 2012. The shift in males predominantly using the Boyes meadow complex, and a change from habitat aggregation to habitat segregation and random association, coincided with females vacating that meadow complex. The shift did not appear to be associated with a pronounced shift in per hectare forage biomass. Also, the change from habitat aggregation to habitat segregation and random habitat associations did not appear to be driven by large groups of males seeking areas with lower grazing pressure. By the time there was a shift to males using Boyes meadow complexes more frequently, male group sizes were less than four in most years.

There are a number of hypotheses of sexual segregation that do not appear to be influential in our data. Climatic variables such as temperature and wind seem unlikely candidates to explain sexual segregation in January in north-coastal California [56, 57]. Daytime and nighttime temperatures were mild for elk [3]. Most often, windspeeds were slight and not cold enough so that large males might be sensitive to inclement conditions [57]. Additionally, past studies have suggested that asynchronous activities between females and males drive segregation because of body size differences [24, 58, 59]. Nevertheless, though ungulates may tend towards individuals of the same sex [60], synchronized activities within each sex probably cannot solely explain sexual segregation [61, 62].

The population was comprised of two female herds before 2011 [35]. The Davison herd appeared to display cyclical abundance during our long-term data collection. This herd peaked in abundance in 1998 and then declined to a record low in 2006 and has since increased to the low 50’s which is approaching the abundance of 1998 [3]. Presumably, the Davison herd is nearing *K* carrying capacity. The Boyes female herd was most abundant early in the study then steadily declined to 4–5 females per year between 2006 and 2010, with no females detected after 2010 [35]. The local extinction of the Boyes herd might have been from vehicle collisions and poaching when this herd was grazing along U.S. Hwy 101 [3]. As such, lethal risks after the local extinction might have been too much for females and precluded recolonization of the Boyes meadow complex. Nonetheless, the lethal risks appeared to not have deterred use by males [32, 33, 37, 38, 63].

Before 2011 the social and habitat segregation patterns might have been affected by the decisions males make when dispersing from the natal herd [64]. If females are plentiful and forage is present in an area then males might settle in that area. The potential mating benefits are not offset by limited forage resources. This scenario might apply to the abundance of females and males when both Boyes and Davison meadow complexes were occupied by females. Males choose Davison over the Boyes meadow complex because in addition to greater per hectare forage there were also more females both during and outside of the mating season. The aggregation of females and males in Davison meadow complex might then result in larger groups of males that were avoided by females.

In a number of years after 2011, male settlement and sexual segregation should seemingly fit the patterns displayed before 2011 when both females herds were present. There were years when per hectare forage was more abundant in the Davison meadow complex and the Davison herd was prevalent. Yet, males used the Boyes meadow complex. In addition to the foraging niches of females and males, another dimension to consider is the level of gregariousness of females and males [26, 27, 65]. Males have a wider forage niche than females which means they could range over a larger area using a wider range of habitats [29]. Females also tend to occur in larger groups and are in closer proximity to one another than are males [27]. Once males came upon the Boyes meadow complex after 2010 they might have been inclined to remain there because of the lower opportunity for encounters with other elk.

Grazing pressure, as measured by per capita forage, was consistently higher in the Davison meadows than in the Boyes meadow complex. A seeming oddity that might have led us to conclude that males were leaving Davison meadows and using the Boyes meadow complex to increase access to forage. Some awareness of lethal risks and measuring per hectare forage biomass made this scenario unlikely. Apparently, grazing pressure was greater in the Davison meadow complex because elk had greater bite sizes and forage intake. In conjunction, the lower grazing pressure in Boyes meadow complex might have been affected by elevated lethal risks which resulted in declining or low elk abundance. If we had measured grazing pressure earlier in the time series when the Boyes herd was most abundant we might have measured more similarity in grazing pressure between the two meadow complexes.

In the Davison meadow complex it seems likely that forage biomass would have been greater in years before 2005, when we began estimating forage abundance. Reed canary grass is found in the Davison meadows and is an invasive species that is unpalatable or has low palatability to elk, particularly in the mature state [35]. Ostensibly, this grass was not apparent early in the time series but became prevalent in later years. It was likely that unpalatable species, like reed canary grass, displaced forbs and grasses that were palatable to elk and prevalent early in the time series [3, 35].

The greater per hecare forage biomass in the Davison meadows indicated that bite size and food intake was greater in the Davison meadows. Low forage biomass in January, which ranged from about 100 to 500 kg^.^ ha^-1^, would in general mean small bite sizes and lower food intake [3]. Nonetheless, forage abundances is positively related to bite size and food intake when forage biomass is 500 kg^.^ ha^-1^ or less [43, 66]. Food intake probably does not begin to plateau or level off until forage abundance is at least 1000 kg^.^ ha^-1^.

What also needs to be pointed out is that the variance in forage biomass in sectors might also influence bite size and food intake. Forage biomass across sectors did not vary nearly as much as the variation within sectors. The considerable small-scale variation in forage biomass indicates that the larger, sector scale estimates of forage biomass are incomplete measures of bite size and forage biomass. It is still feasible to expect that in sectors that had, on average, scarce forage there still might be some places with more forage. This means that males that foraged in the Boyes meadow complex might have found places with greater forage abundances than the average amount, which was what per ha forage expressed.

The considerable small, spatial-scale variation in forage biomass might also be coupled to small scale sexual segregation–social segregation. Males are socially dominant to females and therefore should not be negatively influenced by females in selection of places to forage. At small spatial scales, abundance and variance of forage might also influence male and female distribution. A social mechanism might not be the sole mechanism influencing small scale sexual segregation.

Sexual segregation appears to be founded in intersexual body-size differences which can be coupled to life-history strategies, forage intake, forage niche and social behaviors [1, 9, 10]. Many studies have separated sexual segregation into categories of habitat and social segregation, suggesting males and females segregate at large and small spatial scales by selection of resources or by influences of sociality, respectively. Although the mechanisms that drive changes in social and habitat segregation might differ, there appears to be connections between the two kinds of segregation that remain coupled to size dimorphism. Socially, larger males influence sexual segregation at a small spatial scale, perhaps abundance and variance of forage does as well. At a larger spatial scale, habitat segregation is influenced by size dimorphism that affects the forage niche, vulnerability to predators and, perhaps, the likelihood of encountering conspecific animals. The evidence from our study suggests that when all forage patches are occupied by female elk, social segregation is prevalent. If all forage patches are not occupied by females, segregation and random associations across habitats is a plausible expectation.

## Supporting information

S1 Data Data segregation group size biomass plos one. (XLSX)
